# HLA-B52 allele in giant cell arteritis may indicate diffuse large-vessel vasculitis formation: a retrospective study

**DOI:** 10.1186/s13075-021-02618-4

**Published:** 2021-09-13

**Authors:** Kazuo Kushimoto, Masahiro Ayano, Keisuke Nishimura, Miki Nakano, Yasutaka Kimoto, Hiroki Mitoma, Nobuyuki Ono, Yojiro Arinobu, Koichi Akashi, Takahiko Horiuchi, Hiroaki Niiro

**Affiliations:** 1 Department of Medicine and Biosystemic Science, Kyushu University Graduate School of Medical Sciences, 3-1-1 Maidashi, Higashi-ku, Fukuoka, 812-8582 Japan; 2 Department of Cancer Stem Cell Research, Kyushu University Graduate School of Medical Sciences, Fukuoka, Japan; 3 Department of Endocrinology and Rheumatology, Kurashiki Central Hospital, Kurashiki, Japan; 4 Department of Internal Medicine, Kyushu University Beppu Hospital, Beppu, Japan; 5 Department of Medical Education, Kyushu University Graduate School of Medical Sciences, Fukuoka, Japan

**Keywords:** Large-vessel vasculitis, Giant cell arteritis, Takayasu arteritis, Polymyalgia rheumatica, HLA-B52, PET/CT

## Abstract

**Background:**

This study aimed to identify new characteristics of elderly onset large-vessel vasculitis (EOLVV) by focusing on human leucocyte antigen (HLA) genotype, polymyalgia rheumatica (PMR), and affected vascular lesions observed on positron emission tomography/computed tomography (PET/CT) imaging.

**Methods:**

We retrospectively studied 65 consecutive Japanese patients with large-vessel vasculitis (LVV) who had extracranial vasculitis lesions and underwent PET/CT imaging. PET/CT images were assessed using the semi-quantitative PET visual score of each affected vessel, and the PET vascular activity score (PETVAS) and number of affected vessels were calculated. Subjects were subsequently grouped based on age at onset, superficial temporal artery (STA) involvement, and presence of PMR and compared each group according to HLA genotype. Unsupervised hierarchical cluster analysis was used to identify the patients with similar characteristics in terms of affected vascular lesions detected through PET/CT imaging. The clinical characteristics and PET/CT findings of the population newly identified in this study were examined.

**Results:**

Twenty-seven patients with EOLVV did not meet the American College of Rheumatology 1990 criteria for giant cell arteritis (GCA) and Takayasu arteritis and were considered as unclassified EOLVV (UEOLVV). The unsupervised hierarchical cluster analysis revealed that UEOLVV with PMR and large-vessel GCA (LV-GCA) formed a cluster of LVV with GCA features (i.e., PMR and/or STA involvement) when restricted to patients who were HLA-B52-positive. Patients who were HLA-B52-positive with LVV and GCA features had similar clinical characteristics and patterns of affected vessels and presented with diffuse LVV lesions. HLA-B52-positive patients who had LVV with GCA features also presented with higher PETVAS, more affected vessels, and lower rates of biologics usage and relapse compared to HLA-B52-positive patients with TAK.

**Conclusions:**

Patients who had UEOLVV with PMR had similar characteristics to patients with LV-GCA. Patients who were HLA-B52-positive and had LVV with GCA features presented with diffuse vascular lesions and may comprise a core population of Japanese patients with EOLVV. The findings of HLA-B52 positivity and diffusely affected vessels in patients with EOLVV can be considered as suspicious findings of LV-GCA.

**Supplementary Information:**

The online version contains supplementary material available at 10.1186/s13075-021-02618-4.

## Background

Large-vessel vasculitis (LVV) can be generally categorized into giant cell arteritis (GCA) and Takayasu arteritis (TAK) based on the 2012 Revised International Chapel Hill Consensus Conference Nomenclature of Vasculitides [1]. Both diseases usually differ according to age of onset and distribution of affected vessels. GCA has been classically known as a disease involving vasculitis of the cranial region, particularly the temporal arteries (known as cranial GCA), with an age of onset over 50 years [1, 2]. On the other hand, TAK usually occurs in patients under 50 years; however, elderly onset TAK has also been reported recently [3, 4]. Furthermore, in recent years, large-vessel GCA (LV-GCA), characterized by vascular lesions outside the cranial region, has been widely recognized as a subtype of GCA [2]. Given the different treatment strategies and long-term vascular damage of TAK and GCA, differential diagnosis between both diseases remains important [5, 6]. However, distinguishing LV-GCA without superficial temporal artery (STA) involvement from TAK using conventional classification criteria has been exceedingly difficult [7, 8].

The human leucocyte antigen (HLA) genotype, the presence or absence of polymyalgia rheumatica (PMR), and the distribution of affected vascular lesions are all important in distinguishing between both diseases. HLA-B52 and HLA-DR4 are known characteristic alleles that are associated with TAK and GCA beyond ethnicity, respectively [9, 10]. Ethnic differences in the prevalence of TAK and GCA are also thought to be related to ethnic differences in the distribution of the disease-associated HLA [9, 11, 12]. Therefore, HLA is an important test to distinguish between TAK and GCA. However, a recent study showed that patients with TAK over 40 years had a low prevalence of HLA-B52 [13], and some patients with LV-GCA who had HLA-B52 were also observed in daily practice. From this point of view, classifying LVV using HLA alone is difficult, and it is very important to re-examine the significance of HLA-B52 in combination with other clinical findings in patients with LVV, including elderly onset TAK and LV-GCA. In terms of PMR, a close association between PMR and GCA is well-established; therefore, PMR complications are highly suggestive of GCA [14, 15]. However, according to the current conventional classification criteria of GCA, it is difficult to diagnose LV-GCA in the absence of temporal artery lesions even in the presence of PMR. Moreover, the relationship between PMR and elderly onset LVV (EOLVV) has not yet been elucidated [7].

Imaging is important for diagnosing and evaluating LVV considering the difficulty of diagnostic biopsies in numerous large vascular lesions, as well as the similar histopathological findings of TAK and GCA [2]. Recently, studies have emphasized the usefulness of positron emission tomography/computed tomography (PET/CT) in LVV, which has allowed earlier assessment of inflammatory findings reflecting disease activity over a wider range of vessels prior to the appearance of vascular damage [16, 17]. Accordingly, PET/CT has been used to clarify differences in the distribution of large-vessel lesions in TAK and GCA, subsequently revealing that GCA may have more extensive vascular lesions than TAK, especially in the peripheral extremities [18, 19], in addition to a previous finding that GCA is more prone to STA lesions and axillary artery involvement compared to TAK [1, 5, 20]. Although previous studies have compared PET/CT findings of TAK and GCA, characteristic PET/CT findings of LV-GCA in combination with the HLA genotype have remained unexplored. Since differentiating LV-GCA from elderly onset TAK, which cannot be classified using conventional classification criteria, is difficult, it is important to investigate the new features of EOLVV by combining multiple criteria, such as HLA genotype, presence of PMR, and distribution of affected vessels.

The present study therefore aimed to determine PET/CT findings that can be used to distinguish LV-GCA from TAK and identify new characteristics of LV-GCA related to the HLA genotype, presence of PMR, and affected vascular lesions detected through PET/CT imaging.

## Methods

### Patients and study criteria

A retrospective study was conducted at Kyushu University Hospital and Kurashiki Central Hospital. After differentiating diseases that can cause large-vessel inflammation, such as IgG4-related aortitis, inflammatory aortic aneurysm, infectious aortic aneurysm, syphilitic aortitis, and vascular involvement in Behcet’s disease, a total of 72 consecutive Japanese patients diagnosed with LVV who underwent PET/CT imaging at our department between April 2004 and February 2020 were enrolled herein. After excluding seven patients with isolated cranial GCA, 65 patients with extracranial LVV lesions were ultimately analyzed. LVV with extracranial lesions can be diagnosed based on any of the following criteria: inflammatory findings on PET/CT, vascular wall thickening and damage on CT and magnetic resonance imaging, or postoperative pathological diagnosis. TAK and GCA were classified using the American College of Rheumatology (ACR) 1990 classification criteria [3, 7]. GCA without an evidence of extracranial LVV lesions was defined as isolated cranial GCA based on previous studies [2, 7]. LVV with temporal artery lesions that satisfied the ACR 1990 criteria for GCA was defined as LV-GCA. LVV in patients over 50 years of age that showed no STA involvement and did not fulfill both criteria were considered as unclassified elderly onset LVV (UEOLVV). Patients who had LVV with STA involvement were defined as those who presented any of the following abnormal temporal artery findings upon diagnosis: tenderness on palpation, decreased pulsation, abnormal ultrasound, and positive histological findings. PMR was defined according to Bird’s classification criteria [21]. This study was approved by the ethics committee of Kyushu University Hospital and Kurashiki Central Hospital and was conducted according to the Helsinki Declaration.

### Clinical and laboratory assessment

After retrospectively reviewing patient data, the following information were collected from medical records: age, sex, symptoms, clinical characteristics, laboratory findings, imaging, histological results, medications, and outcomes. Laboratory data, including platelet count, C-reactive protein (CRP), and erythrocyte sedimentation rate (ESR), were collected upon PET/CT imaging in patients with newly diagnosed and untreated LVV. HLA genotypes and anti-phospholipid antibody (aPL) during the clinical course were also determined. The genotyping of HLA-B and HLA-DR allele groups was determined by polymerase chain reaction-reverse sequence specific oligonucleotide method using the Luminex assay system and HLA typing kits. aPL positivity was confirmed based on any positivity for lupus anticoagulants (dilute Russell viper venom time or activated partial thromboplastin time), IgG anti-cardiolipin antibodies (measured using enzyme-linked immunosorbent assay), or IgG anti-cardiolipin-β2 glycoprotein 1 complex antibodies (measured using enzyme-linked immunosorbent assay).

As outcomes, additional biologics usage and disease relapse from their onset to February 2020 (median [interquartile range] years, 6 [3–10] years) were investigated. Disease relapse was defined based on Kerr’s definition for TAK or recurrence of symptoms attributed to active GCA as previously reported [22, 23].

### PET/CT imaging

18F-fluorodeoxyglucose (FDG) PET/CT images were obtained using an integrated PET/CT scanner Discovery STE (GE Medical Systems, Milwaukee, WI, USA) and Biograph mCT. Prior to FDG administration, patients fasted for at least 4 h, after which scans were conducted from the middle of the thigh to the top of the skull 60 min after FDG administration.

### PET/CT interpretation and PET vascular activity score

This study compared PET/CT findings in patients with newly diagnosed and untreated LVV to properly evaluate inflammatory findings without treatment effects. One or two nuclear medicine specialists interpreted the PET/CT images and documented the presence of vasculitis lesions. Two experienced rheumatologists then blindly reviewed the patients’ PET/CT scan and visually determined a semi-quantitative PET visual score on each arterial territory using a pseudo-color spectrum scale (recommended scoring protocol: 0, no FDG uptake; 1, less than liver; 2, equal to liver; 3, greater than liver) [17, 24, 25]. To determine the details regarding inflammatory vascular distribution in patients with LVV, 15 specific extracranial arterial territories (ascending aorta, aortic arch, descending thoracic aorta, abdominal aorta, innominate artery, right/left carotid arteries, right/left subclavian arteries, right/left axially arteries, right/left iliac arteries, and right/left femoral arteries) were evaluated. Affected vessels were defined as those with a PET visual score of ≥ 2. The PET vascular activity score (PETVAS) was calculated based on a previously reported protocol [26] and recorded as the sum of the semi-quantitative PET visual scores of the 15 specific arterial territories, with scores ranging from 0 to 45.

### Statistical analysis

Data were reported as mean and standard deviation, median and interquartile range, or number and percentage. Comparisons between two groups were performed using the Wilcoxon rank sum test for continuous variables and either the chi-square test or Fisher’s exact test for categorical variables. Multiple group comparisons were performed using the Steel–Dwass test. Unsupervised hierarchical cluster analysis with Ward's method was performed using the PET visual score of each of the 15 specific extracranial arterial territories. All analyses were performed using JMP statistical software (version 15.1.0, SAS Institute, Cary, NC), with *P* values < 0.05 indicating statistical significance.

## Results

### Classification flow diagram of this study

**Fig. 1:**
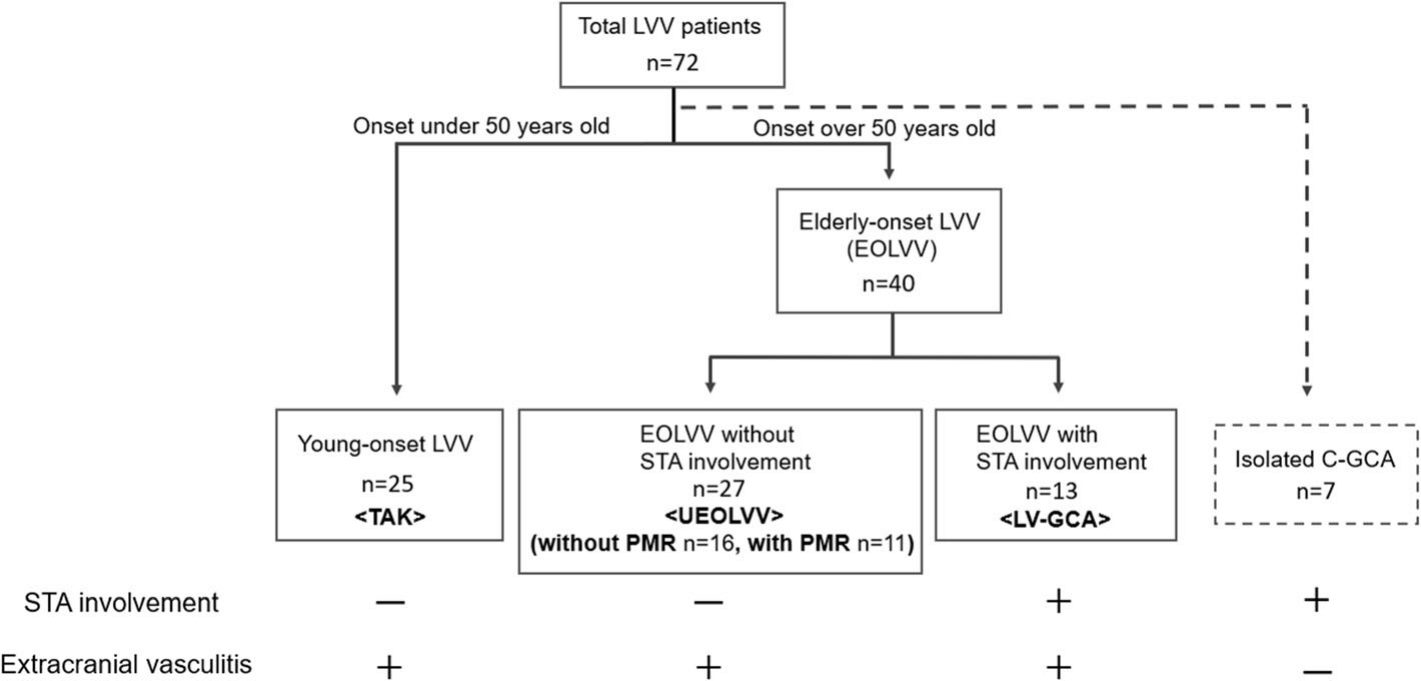
Classification flow diagram. C-GCA, cranial giant cell arteritis; LV-GCA, large-vessel giant cell arteritis; LVV, large-vessel vasculitis; PMR, polymyalgia rheumatica; STA, superficial temporal artery; TAK, Takayasu arteritis; UEOLVV, unclassified EOLVV

A total of 65 patients who had LVV with extracranial LVV lesions were divided into two groups according to age, with a cutoff of 50 years: 25 patients with young-onset LVV diagnosed as TAK and 40 patients with EOLVV. Among the 40 patients with EOLVV, 13 were diagnosed with LV-GCA, whereas the remaining 27 were categorized as UEOLVV (Fig. 1).

### Similar clinical, laboratory, and PET/CT findings between patients who had UEOLVV with PMR and those with LV-GCA

**Fig. 2:**
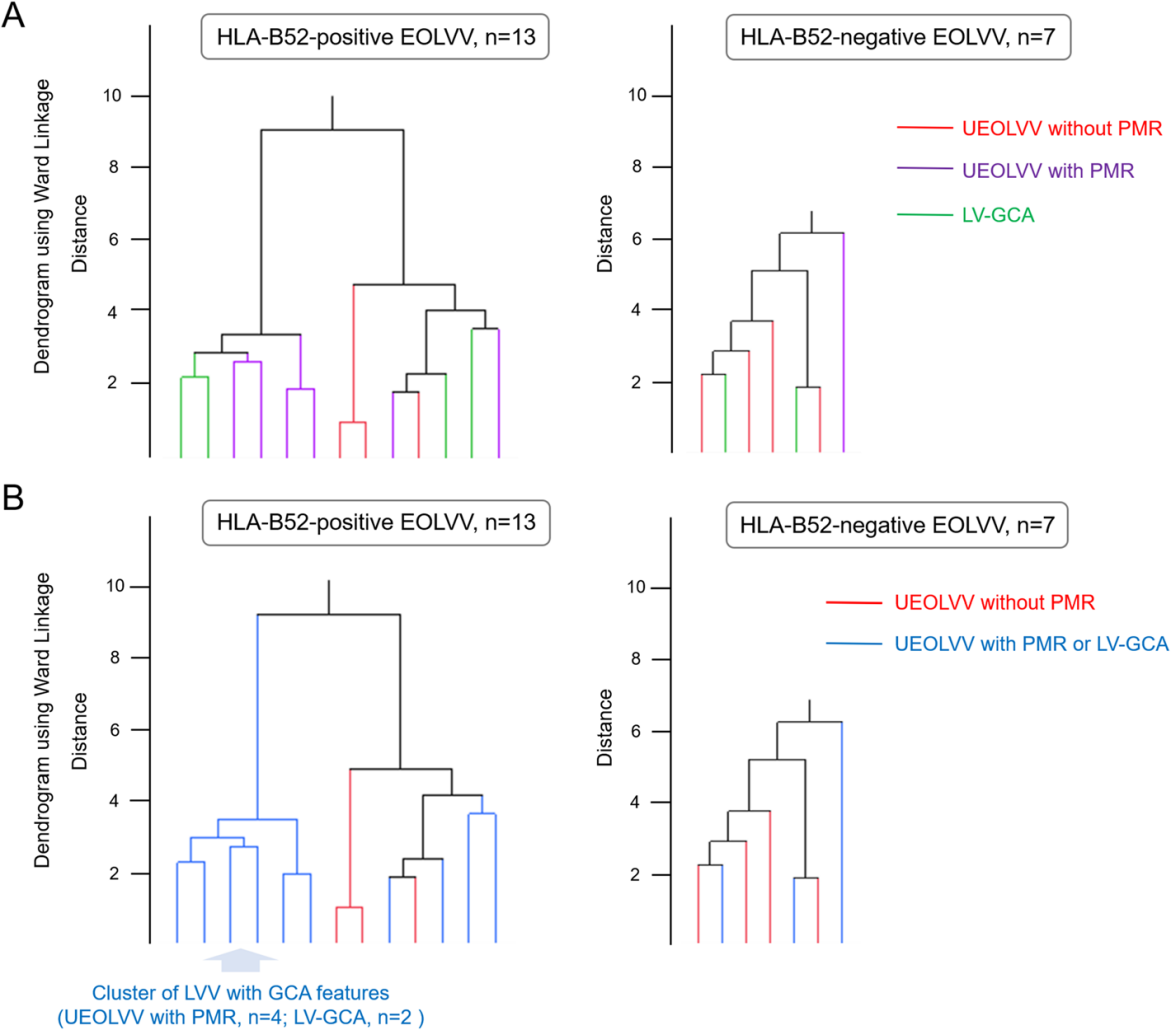
Dendrogram of the unsupervised hierarchical cluster analysis with Ward’s method in patients with EOLVV. **A**, **B** Separate dendrograms of patients who were HLA-B52-positive who had EOLVV and the HLA-B52-negative ones. **A** The red, purple, and green lines represent patients who had UEOLVV without PMR, patients who had UEOLVV with PMR, and patients with LV-GCA, respectively. **B** The red line represents patients who had UEOLVV without PMR, and blue line represents patients who had UEOLVV with PMR or patients with LV-GCA. LVV with GCA features was defined as EOLVV with PMR and/or STA involvement. EOLVV, elderly onset large-vessel vasculitis; GCA, giant cell arteritis; HLA, human leucocyte antigen; LV-GCA, large-vessel GCA; LVV, large-vessel vasculitis; PMR, polymyalgia rheumatica; STA, superficial temporal artery; UEOLVV, unclassified EOLVV

To classify EOLVV based on the HLA genotype, presence of PMR, and affected vascular lesions detected through PET/CT imaging, we initially focused on PMR, subsequently classifying the 27 patients with UEOLVV into two groups: 11 and 16 patients with and without PMR (Fig. 1). Accordingly, patients who had UEOLVV with PMR had higher HLA-B52 positivity and different clinical and laboratory characteristics compared to those without PMR (Table 1). There was no difference in HLA-DR between the two groups. In contrast, patients who had UEOLVV with PMR demonstrated almost similar clinical and laboratory features to those with LV-GCA, except for ESR values (Table 1). Then, unsupervised hierarchical cluster analysis with Ward’s method was performed using the affected vascular lesions detected through PET/CT imaging in patients with newly diagnosed and untreated EOLVV, focusing on HLA-B52, which showed a difference in the frequency among patients with EOLVV. Based on the results, UEOLVV with PMR and LV-GCA was found to form a cluster of LVV with GCA features (i.e., PMR and/or STA involvement) in patients who were HLA-B52-positive. On the other hand, no such clustering was observed in patients who were HLA-B52-negative (Fig. 2). Additionally, the evaluation of the distribution of the affected vessels showed that patients who had UEOLVV with PMR and those with LV-GCA had almost the same number and similar diffuse pattern of affected vessels, including the axillary artery (Supplementary Figure S1 and Table S1). These results suggest that UEOLVV with PMR and LV-GCA may exhibit similar characteristics, especially in patients who were HLA-B52-positive (Fig. 2).

**Table 1:** Clinical and laboratory features of patients with EOLVV

Variable	UEOLVV with PMR (*n* = 11)	UEOLVV without PMR (*n =* 16)	*p* value (vs UEOLVV with PMR)	LV-GCA (*n* = 13)	*p* value (vs UEOLVV with PMR)
Age at onset, years, mean ± SD	67.8 ± 8.6	62.9 ± 10.6	0.12	73.5 ± 9.2	0.10
Female, *n* (%)	9/11 (81.8)	14/16 (87.5)	1	8/13 (61.5)	0.39
Positive temporal artery findings, *n* (%)	0/11 (0)	0/16 (0)		13/13 (100)	
PMR diagnosed, *n* (%)	11/11 (100)	0/16 (0)		6/13 (46.2)	
Jaw claudication, *n* (%)	3/11 (27.3)	0/12 (0)	0.09	2/11 (18.2)	1
Newly diagnosed and untreated patients, *n*	11/11	10/16		11/13	
Laboratory findings					
aPL, *n* (%)	3/7 (42.9)	0/7 (0)	0.19	4/6 (66.7)	0.59
HLA-B52, *n* (%)	6/7 (85.7)	3/10 (30.0)	0.0498	4/6 (66.7)	0.56
HLA-DR4, *n* (%)	2/7 (28.6)	3/8 (37.5)	1	2/5 (40)	1
CRP, mg/dl	9.0 (2.6–13.6)	4.4 (1.04–17.4)	0.65	7.5 (4.9–9.9)	0.95
ESR, mm/h	108 (96–116.5)	79.1 (53.8–91.2)	< 0.01	89.7 (81.8–102.3)	0.09
Platelet count, × 10^4^ cells/μl	47.8 (42.3–54.4)	29.4 (17.6–32.0)	< 0.01	40.3 (29.9–51.1)	0.21

Data are presented as median (IQR) unless otherwise indicated. *aPL* anti-phospholipid antibody, *CRP* C-reactive protein, *EOLVV* elderly onset large-vessel vasculitis, *ESR* erythrocyte sedimentation rate, *HLA* human leucocyte antigen, *LV-GCA* large-vessel GCA, *PMR* polymyalgia rheumatica, *UEOLVV* unclassified elderly-onset large-vessel vasculitis

### Patients who were HLA-B52-positive and had LVV with GCA features showed diffuse PET/CT vasculitis findings that were different from those of patients with TAK who were HLA-B52-positive

**Fig. 3:**
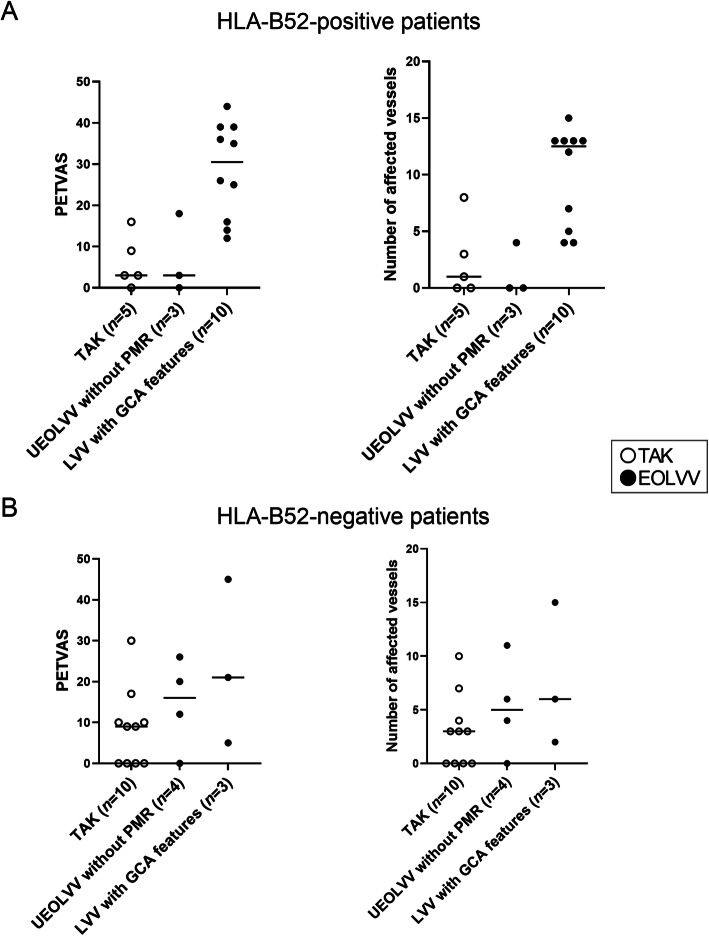
PETVAS and the number of affected vessels in patients with newly diagnosed and untreated LVV. **A**, **B** PETVAS and the number of affected vessels in patients who were HLA-B52-positive (**A**) or HLA-B52-negative (**B**) with LVV were compared between the following three groups: TAK, UEOLVV without PMR, and LVV with GCA features. GCA, giant cell arteritis; HLA, human leucocyte antigen; LVV, large-vessel vasculitis; PETVAS, PET vascular activity score; PMR, polymyalgia rheumatica; TAK, Takayasu arteritis; UEOLVV, unclassified elderly onset LVV

We also compared PET/CT findings between each group, including those with TAK, to assess inflammatory vascular distribution. (Supplementary Figure S2, Fig. 3). Patients with EOLVV had a significantly higher PETVAS and number of affected vessels compared to those with TAK (Supplementary Figure S2). In our analysis, wherein patients with EOLVV were divided into two groups, patients who were HLA-B52-positive who had LVV with GCA features had markedly higher PETVAS and number of affected vessels compared to those with TAK, whereas those who had UEOLVV without PMR showed no significant difference in the same parameters compared to those with TAK (Fig. 3A). Additionally, patients who were HLA-B52-positive who had LVV with GCA features demonstrated higher PET/CT vascular positivity rates in most vascular lesions (including the axillary artery) based on each affected vessel assessment compared to those with TAK (Supplementary Table S2). In contrast, no clear differences were identified between HLA-B52-negative LVV with GCA features and HLA-B52-negative TAK, although the number of inspections was limited (Fig. 3B). These findings indicated that HLA-B52-positive LVV with GCA features presented with more diffuse vascular lesions and formed a distinctly different population from other LVV populations, such as TAK and UEOLVV without PMR, based on the overall assessment of PET/CT vascular inflammation findings.

### HLA-B52-positive patients who had LVV with GCA features exhibited clinical characteristics different from HLA-B52-positive patients with TAK

**Fig. 4:**
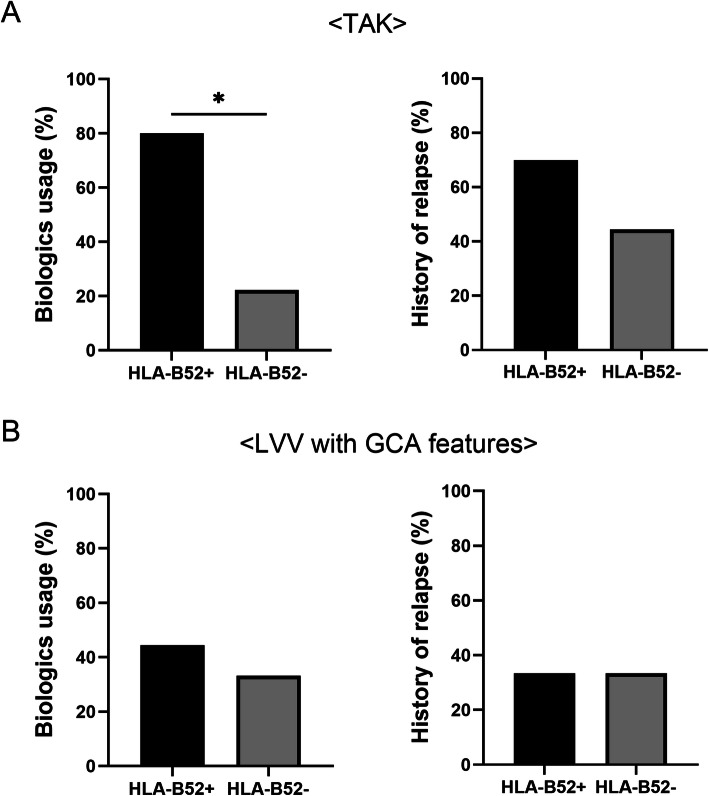
Biologics usage and relapse history of patients with LVV. The biologics usage and relapse history of patients with TAK (**A**) and LVV with GCA features (**B**) were compared between patients who were HLA-B52-positive and -negative. GCA, giant cell arteritis; HLA, human leucocyte antigen; LVV, large-vessel vasculitis; TAK, Takayasu arteritis. *P < 0.05

To explore the clinical characteristics of HLA-B52-positive patients who had LVV with GCA features, the mentioned patients were compared to HLA-B52-positive patients with TAK (Table 2). Compared to HLA-B52-positive patients with TAK, HLA-B52-positive patients who had LVV with GCA features demonstrated higher aPL and HLA-DR4 positivity rates. After also comparing the clinical courses of both diseases, HLA-B52-positive patients who had LVV with GCA features exhibited lower rates of biologics usage and relapse compared to HLA-B52-positive patients with TAK. On the other hand, HLA-B52-positive patients with TAK had a higher rate of biologics usage and relapse compared to HLA-B52-negative ones, whereas no such differences were observed in patients who had EOLVV with GCA features (Fig. 4).

**Table 2:** Clinical and laboratory features of HLA-B52-positive patients with LVV

Variable	TAK, *n* = 10	LVV with GCA features, *n* = 10	*p* value
Age at onset, years, mean ± SD	29.2 ± 11.9	70.1 ± 9.1	< 0.001
Female, *n* (%)	7/10 (70.0)	5/10 (50.0)	0.65
Positive temporal artery findings, *n* (%)	0/10 (0)	4/10 (40.0)	0.09
PMR diagnosed, *n* (%)	0/10 (0)	8/10 (80.0)	< 0.001
Jaw claudication, *n* (%)	0/9 (0)	1/9 (11.1)	1
Biologics usage, *n* (%)	8/10 (80)	4/9 (44.4)	0.17
History of relapse, *n* (%)	7/10 (70)	3/9 (33.3)	0.18
Newly diagnosed and untreated patients, *n*	5/10	10/10	
Laboratory findings			
aPL, *n* (%)	0/4 (0)	4/6 (66.7)	0.08
HLA-B52, *n* (%)	10/10 (100)	10/10 (100)	
HLA-DR4, *n* (%)	0/9 (0)	4/9 (44.4)	0.08
CRP, mg/dl	4.9 (1.8–12.9)	7.5 (4.3–10.8)	0.50
Platelet count, × 10^4^ cells/μl	47.1 (36.8–53.1)	44.1 (35.0–49.5)	0.67

Data are presented as median (IQR) unless otherwise indicated. *aPL* anti-phospholipid antibody, *CRP* C-reactive protein, *GCA* giant cell arteritis, *HLA* human leucocyte antigen, *LVV* large-vessel vasculitis, *PMR* polymyalgia rheumatica, *TAK* Takayasu arteritis

## Discussion

To the best of our knowledge, this has been the first report to elucidate the differences between EOLVV and TAK by focusing on HLA genotype, PMR, and affected vascular lesions detected through PET/CT imaging. Accordingly, our results showed that patients who had LVV with GCA features (i.e., PMR and/or STA involvement), most of whom had HLA-B52, exhibited a significantly increased number of extracranial vascular lesions of LVV compared to those with TAK. The aforementioned findings suggest that HLA-B52-positive LVV with GCA features, which is characteristic to diffuse vasculitis, comprises a core population of patients with EOLVV.

The current study showed that the population of UEOLVV with PMR was very similar to that of LV-GCA (i.e., EOLVV with STA involvement) and distinct from that of UEOLVV without PMR. Patients who had UEOLVV with PMR exhibited similar clinical findings to those with LV-GCA, especially regarding jaw claudication and axillary artery involvement, which are characteristic features of GCA [5, 20]. Additionally, patients who had UEOLVV with PMR and those with LV-GCA had an identifiable pattern of widespread vasculitis lesions in large vessels, unlike patients with TAK, which is consistent with findings presented in several previous reports [19, 27]. Our results suggest that PMR can be as useful a finding as STA involvement for the diagnosis of GCA. In fact, a recent clinical trial utilized PMR as an entry criterion for GCA [23]. Therefore, PMR and STA involvement may be valuable for the diagnosis of GCA.

The present study also demonstrated that patients who had LVV with GCA features demonstrated a higher prevalence of HLA-B52, a disease susceptibility allele of TAK, compared to patients who had UEOLVV without PMR. The findings indicate that HLA-B52 is not a specific finding in patients with TAK and is associated with a population of EOLVV. Among HLA-B52-positive patients, those who had LVV with GCA features exhibited different clinical characteristics, clinical courses, and PET/CT findings compared to those with TAK, indicating that the two diseases are still distinct populations even after limiting our analysis to those with HLA-B52. These findings suggest that the role of HLA-B52 in LVV with GCA features may be different from its role as a disease susceptibility allele in TAK.

HLA-B52-positive patients who had LVV with GCA features uniformly exhibited the most extensively affected vessels based on the number of affected vessels detected on PET/CT. The results of this study suggest that HLA-B52-positive patients with PMR or cranial GCA, which have traditionally been considered overlapping pathologies [14], are likely to develop LVV lesions. A recent study on two large cohorts of LVV showed that the pattern of extensively affected vessels in LVV was more common in the LV-GCA of the Diagnostic and Classification Criteria for Vasculitis cohort, which included patients with various genetic backgrounds, compared to LV-GCA of the North American cohort [19]. Although more detailed examinations of HLA-B52 in these cohorts are needed, we suspect that such differences could be attributed to the carrier rate of HLA-B52. In addition, a survey of HLA in patients with abdominal aortic aneurysms in Japan pointed out an association with MHC class I and reported that HLA-B52 was more common in patients with abdominal aortic aneurysms who had aorto-iliac occlusive disease than those who did not [28]. Our results and these findings indicate that HLA-B52 may be involved in the distribution of affected vessels. In this point, the concept of MHC-I-opathy has recently been proposed for diseases, such as spondyloarthritis and Behcet’s disease. This is a concept of disease in which clinical features of disease localization are shared by specific MHC class I molecules and corresponding tissue antigen structures in the target tissues at sites of contact between the body and the external environment (oral mucosa, gut, and skin) or sites of physical stress, such as entheses, including the mini-entheses in the eye, vessel walls, and valve regions [29]. Given that this disease concept may also be applied to HLA-B52-positive LVV, the distribution of the corresponding antigen of HLA-B52 may play an important role in the formation of vascular lesions outside the cranial region, suggesting that when elderly patients are exposed to chronic inflammatory conditions, such as atherosclerosis, PMR, or GCA, patients who are HLA-B52-positive may be susceptible to inflammation in the extracranial LVV lesions and may be more likely to develop vascular lesions. Further investigations on the mechanisms underlying the high prevalence of HLA-B52 in patients with LVV with GCA features and the wide range of affected vascular lesions in HLA-B52-positive population are needed. Our results and findings indicate that HLA-B52 may be involved in the pathogenesis of diffuse LVV complicated with PMR and cranial GCA.

This study showed that patients who were HLA-B52-positive and had LVV with GCA features exhibited lower rates of biologics usage and relapse compared to patients who were HLA-B52-positive with TAK, who were more also likely to be refractory to treatment or experience relapse compared to the HLA-B52-negative ones. This was consistent with previous reports [30], whereas patients who were HLA-B52-positive with GCA showed no obvious differences in clinical course compared to the HLA-B52-negative ones. These results suggest that there is a difference in therapeutic response between patients who were HLA-B52-positive with TAK and patients who were HLA-B52-positive and had LVV with GCA features. Unlike TAK, HLA-B52 is possibly not involved in treatment resistance in GCA. Previous studies reported that patients with diffuse vasculitis lesions have shown less vascular damage [19], whereas LV-GCA is more likely to cause vasodilatation and form dissecting lesions, unlike TAK, which tends to form stenotic lesions [6]. These findings indicate that HLA-B52 may be involved not only in the distribution of affected vascular lesions but also in the severity of vascular inflammation and damage, which may result in differences in treatment response between LV-GCA and TAK.

The present study showed that among patients with UEOLVV, those with PMR exhibited more diffuse vascular lesions compared to those without PMR, which is remarkable in HLA-B52 carriers. PET/CT can possibly be used to identify the potential presence of LVV lesions [31]. Reports have shown that imaging can detect coexisting subclinical LVV lesions in patients diagnosed with PMR [32]. In clinical settings, complicated extracranial LVV lesions are difficult to determine in patients with PMR based on clinical findings alone. As such, patients with PMR, especially those with HLA-B52, may need to undergo PET/CT to examine the coexistence of LVV early in the course of PMR.

The current study has some limitations worth noting. First, this study had a small sample size and was limited to Japanese patients. Thus, our results need to be verified in a multiracial study with a larger sample size. Second, this was a retrospective study, with lacking HLA measurements and a potential for hindsight bias. The treatment course was also studied backward, and treatment selection was left to the discretion of each attending physician. As such, further prospective studies are needed. Third, the mechanism by which HLA-B52 is involved in the pathogenesis of diffuse LVV with GCA features also needs to be investigated in the future. Lastly, long-term outcomes, such as vascular damage were not assessed. Further studies on the distribution of vasculitis and vascular damage may help predict future vascular damage in LVV.

## Conclusions

The current study showed that patients who had EOLVV without STA involvement but with PMR demonstrated similar patient backgrounds and vessel distributions (observed via PET/CT) to patients with LV-GCA who satisfied the 1990 ACR classification criteria. Moreover, HLA-B52-positive patients who had EOLVV with PMR and/or STA involvement presented with diffuse vascular lesions and may comprise a core population for LV-GCA in among Japanese individuals. Thus, the findings of HLA-B52-positivity and diffusely affected vessels, as well as those of PMR, in patients with EOLVV can be considered as suspicious findings of LV-GCA.

## Supplementary Information

**Additional file 1.**

## Data Availability

All data generated or analyzed during this study are included in this published article and its supplementary information files.

## Abbreviations

ACR: American College of Rheumatology
aPL: Anti-phospholipid antibody
CRP: C-reactive protein
EOLVV: Elderly onset large-vessel vasculitis
ESR: Erythrocyte sedimentation rate
FDG: Fluorodeoxyglucose
GCA: Giant cell arteritis
HLA: Human leucocyte antigen
LV-GCA: Large-vessel GCA
LVV: Large-vessel vasculitis
PET/CT: Positron emission tomography/computed tomography
PETVAS: PET vascular activity score
PMR: Polymyalgia rheumatica
STA: Superficial temporal artery
TAK: Takayasu arteritis
UEOLVV: Unclassified elderly onset LVV

## References

[1] Jennette JC, Falk RJ, Bacon PA, Basu N, Cid MC, Ferrario F (2013). 2012 revised International Chapel Hill Consensus Conference Nomenclature of Vasculitides. Arthritis Rheum.

[2] Lensen KD, Voskuyl AE, Comans EF, van der Laken CJ, Smulders YM (2016). Extracranial giant cell arteritis: A narrative review. Neth J Med.

[3] Arend WP, Michel BA, Bloch DA, Hunder GG, Calabrese LH, Edworthy SM (1990). The American College of Rheumatology 1990 criteria for the classification of Takayasu arteritis. Arthritis Rheum.

[4] Watanabe Y, Miyata T, Tanemoto K (2015). Current clinical features of new patients with Takayasu arteritis observed from cross-country research in Japan: age and sex specificity. Circulation..

[5] Kermani TA (2019). Takayasu arteritis and giant cell arteritis: are they a spectrum of the same disease?. Int J Rheum Dis.

[6] Tombetti E, Godi C, Ambrosi A, Doyle F, Jacobs A, Kiprianos AP (2018). Novel angiographic scores for evaluation of large vessel vasculitis. Sci Rep.

[7] Hunder GG, Bloch DA, Michel BA, Stevens MB, Arend WP, Calabrese LH (1990). The American College of Rheumatology 1990 criteria for the classification of giant cell arteritis. Arthritis Rheum.

[8] Muratore F, Kermani TA, Crowson CS, Green AB, Salvarani C, Matteson EL (2015). Large-vessel giant cell arteritis: a cohort study. Rheumatology (Oxford).

[9] Terao C (2016). Revisited HLA and non-HLA genetics of Takayasu arteritis--where are we?. J Hum Genet.

[10] Jacobsen S, Baslund B, Madsen HO, Tvede N, Svejgaard A, Garred P (2002). Mannose-binding lectin variant alleles and HLA-DR4 alleles are associated with giant cell arteritis. J Rheumatol.

[11] Kimura A, Kitamura H, Date Y, Numano F (1996). Comprehensive analysis of HLA genes in Takayasu arteritis in Japan. Int J Cardiol..

[12] Mackie SL, Taylor JC, Haroon-Rashid L, Martin S, Dasgupta B, Gough A (2015). Association of HLA-DRB1 amino acid residues with giant cell arteritis: genetic association study, meta-analysis and geo-epidemiological investigation. Arthritis Res Ther..

[13] Sahin Z, Bıcakcıgil M, Aksu K, Kamali S, Akar S, Onen F (2012). Takayasu’s arteritis is associated with HLA-B* 52, but not with HLA-B* 51, in Turkey. Arthritis Res Ther..

[14] Dejaco C, Duftner C, Buttgereit F, Matteson EL, Dasgupta B (2017). The spectrum of giant cell arteritis and polymyalgia rheumatica: revisiting the concept of the disease. Rheumatology (Oxford).

[15] Cantini F, Niccoli L, Storri L, Nannini C, Olivieri I, Padula A (2004). Are polymyalgia rheumatica and giant cell arteritis the same disease?. Semin Arthritis Rheum.

[16] Pelletier-Galarneau M, Ruddy TD (2019). PET/CT for diagnosis and management of large-vessel vasculitis. Curr Cardiol Rep.

[17] Walter MA, Melzer RA, Schindler C, Müller-Brand J, Tyndall A, Nitzsche EU (2005). The value of [18F]FDG-PET in the diagnosis of large-vessel vasculitis and the assessment of activity and extent of disease. Eur J Nucl Med Mol Imaging.

[18] Prieto-González S, Depetris M, García-Martínez A, Espígol-Frigolé G, Tavera-Bahillo I, Corbera-Bellata M (2014). Positron emission tomography assessment of large vessel inflammation in patients with newly diagnosed, biopsy-proven giant cell arteritis: a prospective, case-control study. Ann Rheum Dis.

[19] Gribbons KB, Ponte C, Carette S, Craven A, Cuthbertson D, Hoffman GS (2020). Patterns of arterial disease in Takayasu arteritis and giant cell arteritis. Arthritis Care Res (Hoboken).

[20] Schmidt WA, Seifert A, Gromnica-Ihle E, Krause A, Natusch A (2008). Ultrasound of proximal upper extremity arteries to increase the diagnostic yield in large-vessel giant cell arteritis. Rheumatology (Oxford).

[21] Bird HA, Esselinckx W, Dixon AS, Mowat AG, Wood PH (1979). An evaluation of criteria for polymyalgia rheumatica. Ann Rheum Dis.

[22] Kerr GS, Hallahan CW, Giordano J, Leavitt RY, Fauci AS, Rottem M (1994). Takayasu arteritis. Ann Intern Med.

[23] Unizony SH, Dasgupta B, Fisheleva E, Rowell L, Schett G, Spiera R (2013). Design of the tocilizumab in giant cell arteritis trial. Int J Rheumatol.

[24] Meller J, Strutz F, Siefker U, Scheel A, Sahlmann CO, Lehmann K (2003). Early diagnosis and follow-up of aortitis with [(18)F]FDG PET and MRI. Eur J Nucl Med Mol Imaging.

[25] Kang F, Han Q, Zhou X, Zheng Z, Wang S, Ma W (2020). Performance of the PET vascular activity score (PETVAS) for qualitative and quantitative assessment of inflammatory activity in Takayasu’s arteritis patients. Eur J Nucl Med Mol Imaging.

[26] Grayson PC, Alehashemi S, Bagheri AA, Civelek AC, Cupps TR, Kaplan MJ (2018). 18 F-fluorodeoxyglucose-positron emission tomography as an imaging biomarker in a prospective, longitudinal cohort of patients with large vessel vasculitis. Arthritis Rheumatol.

[27] Power SP, O'Mahony D (2019). Diffuse large vessel giant cell arteritis found by 18Fluorodeoxyglucose PET/CT imaging. Lancet.

[28] Sugimoto T, Sada M, Miyamoto T, Yao H (2003). Genetic analysis on HLA loci in Japanese patients with abdominal aortic aneurysm. Eur J Vasc Endovasc Surg..

[29] McGonagle D, Aydin SZ, Gül A, Mahr A, Direskeneli H (2015). ‘MHC-I-opathy’-unified concept for spondyloarthritis and Behçet disease. Nat Rev Rheumatol..

[30] Ohigashi H, Tamura N, Ebana Y, Harigai M, Maejima Y, Ashikaga T (2017). Effects of immunosuppressive and biological agents on refractory Takayasu arteritis patients unresponsive to glucocorticoid treatment. J Cardiol.

[31] Blockmans D, de Ceuninck L, Vanderschueren S, Knockaert D, Mortelmans L, Bobbaers H (2006). Repetitive 18F-fluorodeoxyglucose positron emission tomography in giant cell arteritis: a prospective study of 35 patients. Arthritis Rheum.

[32] González-Gay MA, Matteson EL, Castañeda S (2017). Polymyalgia rheumatica. Lancet.

