# Supplementary material for: HLA-B52 allele in giant cell arteritis may indicate diffuse large-vessel vasculitis formation: a retrospective study
Source: Arthritis Res Ther. 2021 Sep 13;23:238. doi: 10.1186/s13075-021-02618-4 (PMC8436550; doi:10.1186/s13075-021-02618-4)
Supplement: Supplementary file 1 — Additional file 1. [file 13075_2021_2618_MOESM1_ESM.pdf]

SUPPLEMENTARY MATERIAL

Supplementary Table S1. PET/CT vascular positivity rate in newly diagnosed and untreated patients with EOLVV

Valiable	UEOLVV with PMR (*n*=11)	UEOLVV without PMR (*n*=10)	*p* value (vs UEOLVV with PMR)	LV-GCA (*n*=11)	*p* value (vs UEOLVV with PMR)
Common carotid (left, right), %	72.7, 81.8	30.0, 20.0	0.09, <0.01	45.5, 45.5	0.39, 0.18
Ascending Ao, %	81.8	30.0	0.03	54.6	0.36
Arch, %	81.8	60.0	0.36	72.7	1
Descending Thoracic Ao, %	90.9	60.0	0.15	63.6	0.31
Abdominal Ao, %	90.9	60.0	0.15	81.8	1
Innominate, %	54.6	60.0	1	63.6	1
Subclavian (left, right), %	63.6, 72.7	40.0, 50.0	0.39, 0.39	81.8, 72.7	0.64, 1
Axillary (left, right), %	54.6, 72.7	30.0, 30.0	0.39, 0.09	54.6, 54.6	1, 0.66
Iliac (left, right), %	63.6, 63.6	30.0, 30.0	0.20, 0.20	45.5, 45.5	0.67, 0.67
Femoral (left, right), %	36.4, 54.6	10.0, 20.0	0.31, 0.18	36.4, 45.5	1, 1

EOLVV, elderly onset large-vessel vasculitis; LV-GCA, large-vessel GCA; PET/CT, positron emission tomography/computed tomography; PMR, polymyalgia rheumatica; UEOLVV, unclassified elderly-onset large vessel vasculitis.

Supplementary Table S2. PET/CT vascular positivity rate in newly diagnosed and untreated HLA-B52-positive patients with LVV

Valiable	TAK, *n*=5	LVV with GCA features, *n*=10	*p* value
Common carotid (left, right), %	20, 20	60, 70	0.28, 0.12
Ascending Ao, %	20	70	0.12
Arch, %	20	90	0.02
Descending Thoracic Ao, %	0	80	<0.01
Abdominal Ao, %	40	100	0.02
Innominate, %	0	60	0.04
Subclavian (left, right), %	20, 20	70, 60	0.12, 0.28
Axillary (left, right), %	20, 0	70, 70	0.12, 0.03
Iliac (left, right), %	20, 0	60, 50	0.28, 0.10
Femoral (left, right), %	20, 20	30, 50	1, 0.58

GCA, giant cell arteritis; HLA, human leucocyte antigen; LVV, large-vessel vasculitis; PET/CT, positron emission tomography/computed tomography; TAK, Takayasu arteritis.

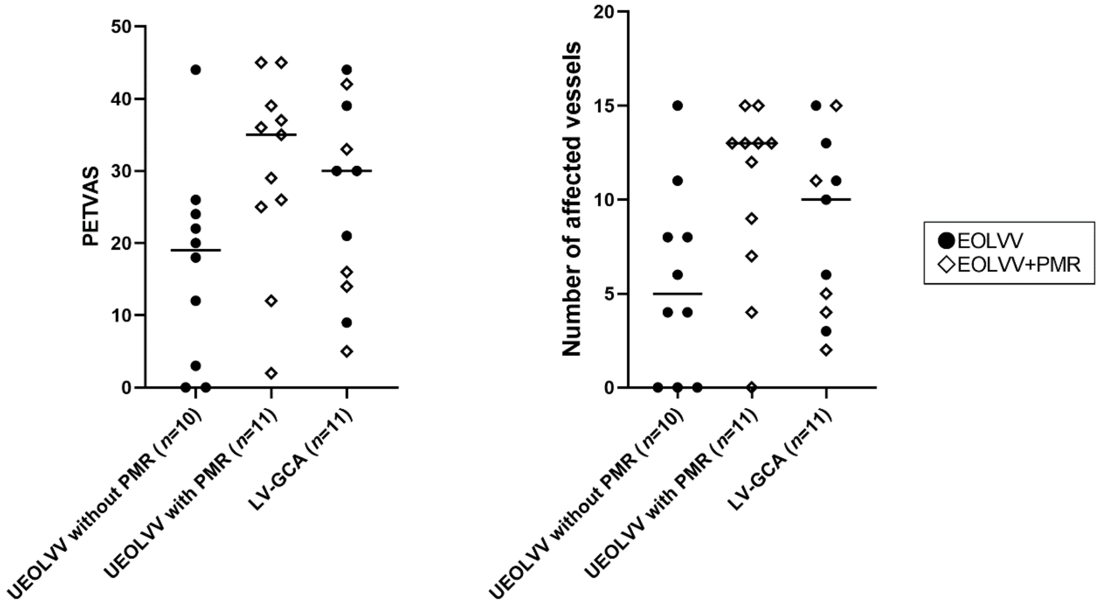

Supplementary Figure S1. PETVAS and the number of affected vessels in patients with newly diagnosed and untreated EOLVV

PETVAS and the number of affected vessels were compared between the following three groups: UEOLVV without PMR, UEOLVV with PMR, and LV-GCA.

EOLVV, elderly onset large-vessel vasculitis; LV-GCA, large-vessel GCA; PETVAS, positron emission tomography vascular activity score; PMR, polymyalgia rheumatica; UEOLVV, unclassified EOLVV.

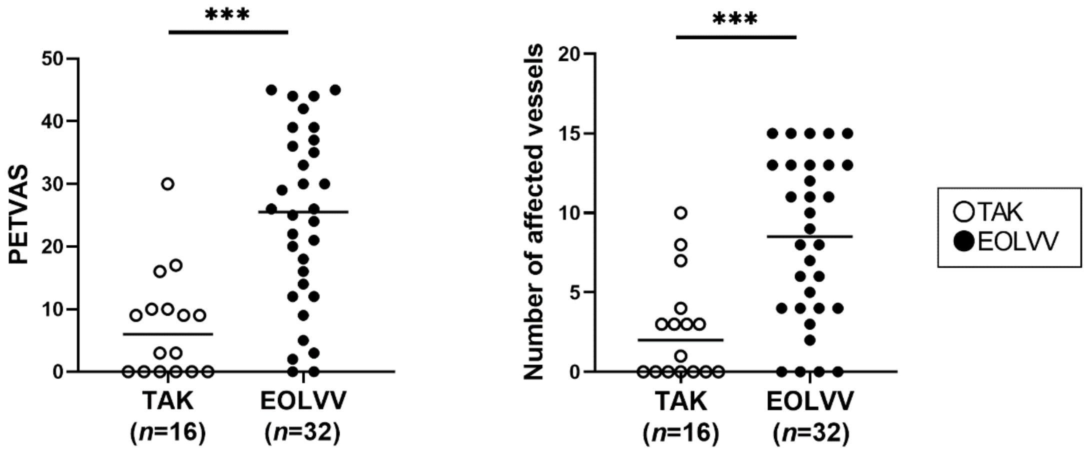

Supplementary Figure S2. PETVAS and the number of affected vessels in patients with newly diagnosed and untreated LVV

PETVAS and the number of affected vessels were compared between patients with TAK and EOLVV.

EOLVV, elderly onset large-vessel vasculitis; LVV, large-vessel vasculitis; PETVAS, positron emission tomography vascular activity score; TAK, Takayasu arteritis. ***P < 0.001
